# Enabling Precision Medicine for Rare Head and Neck Tumors: The Example of BRAF/MEK Targeting in Patients With Metastatic Ameloblastoma

**DOI:** 10.3389/fonc.2019.01204

**Published:** 2019-11-12

**Authors:** Maxime Brunet, Emmanuel Khalifa, Antoine Italiano

**Affiliations:** ^1^Department of Medicine, Institut Bergonié, Bordeaux, France; ^2^Department of Tumor Genetics, Institut Bergonié, Bordeaux, France; ^3^Faculty of Médicine, University of Bordeaux, Bordeaux, France; ^4^INSERM, ACTION U1218, Bordeaux, France

**Keywords:** ameloblastoma, BRAF, targeted therapeutic drugs, rare tumor, precision oncology

## Abstract

**Background:** Ameloblastoma is a rare head and neck tumor characterized by a high incidence of BRAF mutation providing a rationale for the use of BRAF inhibitors in patients with advanced disease.

**Methods:** We report the case of a 26-year old female presenting with metastatic ameloblastoma. A molecular screening of the tumor revealed a BRAF V600E mutation.

**Results:** The patient started treatment with dabrafenib and trametinib and experienced complete response which is still ongoing 30 weeks after treatment onset.

**Conclusions:** The complete response observed here illustrate the role of molecular profiling in complicate clinical situation of rare head and neck cancer and the potential benefit of BRAF-targeted therapy in ameloblastoma carrying *BRAF* V600E mutation.

## Introduction

Precision oncology aims to tailor the therapeutic strategy based on tumor's molecular alterations. Previous studies have suggested the validity of such approach in rare and ultra-rare tumor subtypes, including rare head and neck cancers; with the identification of targetable alterations in up to 93% of cases (1). Ameloblastomas are rare tumors, which often have a benign course. Metastatic evolution is extremely rare and there is no standard of care for patients in this setting.

## Case Report

In November 2018, a 26-year old female was referred for hemoptysis and cough of several weeks' duration. Her past medical history was significant for the diagnosis, at the age of 13 years, of an ameloblastoma of the right mandible (tooth 48) treated by surgery with clear margins and no evidence of locoregional relapse since then. A CT scan was performed which showed numerous bilateral nodules. The patient underwent a CT-guided percutaneous lung biopsy of one of these nodules. Histological examination was in favor of lung metastasis of ameloblastoma.

**Figure 1:**
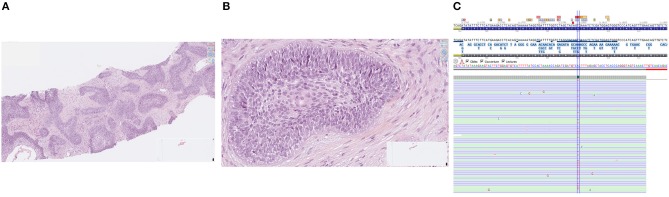
Histological features of ameloblastoma (**A**: low magnification, **B**: high magnification) and BRAF V600E gene mutation assessed by next-generation sequencing **(C)**.

**Figure 2:**
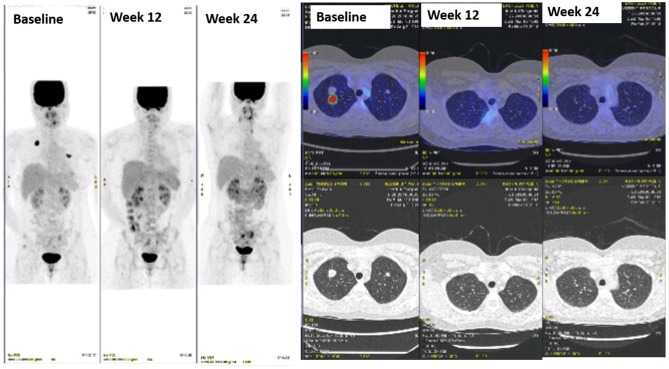
Sustained complete response to dabrafenib + trametinib in a patient with ameloblastoma metastatic to the lungs.

The patient was enrolled in our molecular screening program after signature of informed consent (Bergonié Institut Profiling study, NCT02534649) and gave also written informed consent for the publication of this case report. Next-generation sequencing of the metastatic tumor sample was performed as previously described and revealed the presence of a *BRAF* gene V600E mutation [Figure 1; (2)]. Given the lack of validated standard treatment for advanced ameloblastoma, the molecular tumor board recommended initiation of a BRAF-targeted therapy. The patient started treatment with dabrafenib (150 mg BID) combined with trametinib (2 mg QD). The first TEP-CT scan performed 12 weeks after treatment onset showed competed response according to RECIST and PERCIST criteria (Figure 2). The patient is still doing well and in complete remission 30 weeks after treatment initiation.

## Discussion

The ameloblastoma is a histologically almost always benign odontogenic tumor of the jaw bones with a potential of locoregional recurrence (3). Distant metastases are extremely rare.

Anecdotal response has been reported with platinum salts agents but there is no standard of care in this setting (4). A seminal study investigating the molecular landscape of ameloblastomas revealed that ameloblastomas are characterized by the a frequent incidence of BRAF V600E mutations particularly in tumors located in the mandibular region while mutations of the SMO gene (39%) were predominant in maxillary tumors (82%) (5). This study and others provided a rationale to investigate BRAF targeting in patients with advance ameloblastoma. Previous case reports have suggested the clinical activity of BRAF inhibitor such as vemurafenib in patients with advanced ameloblastoma (Table 1). We opted here for the combination of a BRAF and MEK inhibitors. Indeed, several studies performed in melanomas, and other BRAF-driven malignancy, showed that acquired resistance to BRAF inhibitors frequently develops through reactivation of the mitogen-activated protein kinase (MAPK) pathway, resulting in limited progression-free survival (10) In addition, the use of BRAF inhibitors as single agent may induce the development of secondary skin tumors, originating from a paradoxical activation of the MAPK pathway in cells without a *BRAF* mutation (10). In melanomas, combining a BRAF inhibitor with a MEK inhibitor addresses the limitations of single-agent BRAF inhibitors and results in a significant delay in the emergence of resistance, with a longer overall than with BRAF inhibitor alone, as well as a decreased incidence of BRAF-inhibitor–induced skin tumors (10). The toxicity of such a combination profile is generally good with the most common adverse events being rash, pyrexia, asthenia, headache, nausea, and arthralgia (11). The complete response observed here illustrate the role of molecular profiling in complicate clinical situation of rare head and neck cancer and the potential benefit of BRAF-targeted therapy in ameloblastoma carrying *BRAF* V600E mutation.

**Table 1:** Clinical reports of BRAF-targeting in advanced ameloblastoma.

**References**	**Clinical setting**	**Tumor stage**	**Treatment**	**Outcome**
Fernandes et al. (6)	29 years old female	Locally advanced	Vemurafenib	Partial response PFS not reached at 11 months after treatment onset
Tan et al. (7)	85 years old male	Locally advanced	Dabrafenib	Partial response
Faden and Algazi (8)	83 years old male	Locally advanced	Dabrafenib	Partial response PFS not reached at 12 months
Kaye et al. (9)	40 years old male	Metastatic	Dabrafenib + trametinib	Partial response Not reached at 5 months after treatment onset

## Data Availability Statement

All datasets generated for this study are included in the article/supplementary material.

## Ethics Statement

This study was approved by the institutional review board of Institut Bergonié.

## Author Contributions

AI: conception and design. EK and AI: collection and assembly of data. All authors: data analysis, interpretation, manuscript writing, and final approval of manuscript.

### Conflict of Interest

The authors declare that the research was conducted in the absence of any commercial or financial relationships that could be construed as a potential conflict of interest.

## References

[1] Kato S Kurasaki K Ikeda S Kurzrock R. Rare tumor clinic: the University of California San Diego Moores Cancer Center experience with a precision therapy approach. Oncologist. (2018) 23:171–8. 10.1634/theoncologist.2017-0199 29038235 PMC5813742

[2] Cousin S Grellety T Toulmonde M Auzanneau C Khalifa E Laizet Y. Clinical impact of extensive molecular profiling in advanced cancer patients. J Hematol Oncol. (2017) 10:45. 10.1186/s13045-017-0411-5 28179005 PMC5299780

[3] Sciubba J Odontogenic tumors. In: Barnes L Eveson J Reichart P Sidransky D, editors. World Health Organization Classification of Tumors, Pathology and Genetics of Head and Neck Tumors. Lyon: IARC Press (2005). p. 287–93.

[4] McClary AC West RB McClary AC Pollack JR Fischbein NJ Holsinger CF. Ameloblastoma: a clinical review and trends in management. Eur Arch Otorhinolaryngol. (2016) 273:1649–61. 10.1007/s00405-015-3631-8 25926124

[5] Sweeney RT McClary AC Myers BR Biscocho J Neahring L Kwei KA. Identification of recurrent SMO and BRAF mutations in ameloblastomas. Nat Genet. (2014) 46:722–5. 10.1038/ng.2986 24859340 PMC4418232

[6] Fernandes GS Girardi DM Bernardes JPG Fonseca FP Fregnani ER. Clinical benefit and radiological response with BRAF inhibitor in a patient with recurrent ameloblastoma harboring V600E mutation. BMC Cancer. (2018) 18:887. 10.1186/s12885-018-4802-y 30208863 PMC6134697

[7] Tan S Pollack JR Kaplan MJ Colevas AD West RB. BRAF inhibitor treatment of primary BRAF-mutant ameloblastoma with pathologic assessment of response. Oral Surg Oral Med Oral Pathol Oral Radiol. (2016) 122:e5–7. 10.1016/j.oooo.2015.12.016 27209484

[8] Faden DL Algazi A. Durable treatment of ameloblastoma with single agent BRAFi Re: Clinical and radiographic response with combined BRAF-targeted therapy in stage 4 ameloblastoma. J Natl Cancer Inst. (2016) 109:djw190. 10.1093/jnci/djw190 27671684

[9] Kaye FJ Ivey AM Drane WE Mendenhall WM Allan RW. Clinical and radiographic response with combined BRAF-targeted therapy in stage 4 ameloblastoma. J Natl Cancer Inst. (2014) 107:378. 10.1093/jnci/dju378 25475564

[10] Torres-Collado AX Knott J Jazirehi AR. Reversal of resistance in targeted therapy of metastatic melanoma: lessons learned from vemurafenib (BRAF^V600E^-Specific Inhibitor). Cancers. (2018) 10:E157. 10.3390/cancers10060157 29795041 PMC6025215

[11] Knispel S Zimmer L Kanaki T Ugurel S Schadendorf D Livingstone E. The safety and efficacy of dabrafenib and trametinib for the treatment of melanoma. Expert Opin Drug Saf . (2018) 17:73–87. 10.1080/14740338.2018.1390562 29050517

